# Assessment of Nonradioactive Multispectral Optoacoustic Tomographic Imaging With Conventional Lymphoscintigraphic Imaging for Sentinel Lymph Node Biopsy in Melanoma

**DOI:** 10.1001/jamanetworkopen.2019.9020

**Published:** 2019-08-14

**Authors:** Ingo Stoffels, Philipp Jansen, Maximilian Petri, Lukas Goerdt, Titus J. Brinker, Klaus G. Griewank, Thorsten D. Poeppel, Dirk Schadendorf, Joachim Klode

**Affiliations:** 1 Department of Dermatology, Venerology and Allergology, University Hospital-Essen, University of Duisburg-Essen, Essen, Germany; 2 West German Cancer Center, University Duisburg-Essen, Essen, Germany; 3 German Consortium for Translational Cancer Research, Partner Site, University Hospital-Essen, Essen, Germany; 4 Department of Nuclear Medicine, University Essen-Duisburg, University of Duisburg, Essen, Germany

## Abstract

**Importance:**

The metastatic status of sentinel lymph nodes (SLNs) is the most relevant prognostic factor in breast cancer, melanoma, and other tumors. The conventional standard to label SLNs is lymphoscintigraphy with technetium Tc 99m. A worldwide shortage and known disadvantages of Tc 99m have intensified efforts to establish alternative, nonradioactive imaging techniques.

**Objective:**

To assess a new nonradioactive method using multispectral optoacoustic tomographic (MSOT) imaging in comparison with conventional lymphoscintigraphic imaging for SLN biopsy (SLNB) in melanoma.

**Design, Setting, and Participants:**

Analysis of a cross-sectional study was conducted at the University Hospital-Essen, Skin Cancer Center, Essen, Germany. Between June 2, 2014, and February 22, 2019, 83 patients underwent SLNB with an additional preoperative indocyanine green (ICG) application. Sentinel lymph node basins were preoperatively identified by MSOT imaging, and ICG-labeled SLNs were intraoperatively detected using a near-infrared camera. The surgeons were blinded to the lymphoscintigraphic imaging results in the beginning of the SLNB. Use of a γ probe was restricted until the SLNB procedure was attempted by the nonradioactive method.

**Main Outcomes and Measures:**

Concordance of SLN basins and SLNs identified by MSOT imaging plus near-infrared camera vs lymphoscintigraphic imaging plus single-photon emission computed tomographic or computed tomographic imaging was assessed.

**Results:**

Of the 83 patients (mean [SD] age, 54.61 [17.53] years), 47 (56.6%) were men. In 83 surgical procedures, 165 SLNs were excised. The concordance rate of ICG-labeled and Tc 99m–marked detected SLN basins was 94.6% (n = 106 of 112). Intraoperatively, 159 SLNs were detected using a near-infrared camera and 165 were detected by a γ probe, resulting in a concordance rate of 96.4%. Multispectral optoacoustic tomographic imaging visualized SLNs in all anatomic regions with high penetration depth (5 cm).

**Conclusions and Relevance:**

The findings of this study suggest that nonradioactive SLN detection via MSOT imaging allows identification of SLNs at a frequency equivalent to that of the current radiotracer conventional standard. Multispectral optoacoustic tomographic imaging appears to be a viable nonradioactive alternative to detect SLNs in malignant tumors.

## Key Points

**Question:**

Is a nonradioactive imaging approach for sentinel lymph node detection a viable alternative to radioactive technetium?

**Findings:**

In this cross-sectional study of 83 patients with newly diagnosed melanoma, sentinel lymph node detection via indocyanine green and multispectral optoacoustic tomographic imaging was concordant with the detection frequency of conventional lymphoscintigraphic imaging with technetium Tc 99m.

**Meaning:**

Multispectral optoacoustic tomographic imaging may allow nonradioactive detection of sentinel lymph nodes at a frequency similar to the current radiotracer standard.

## Teaser

This cross-sectional study evaluates use of nonradioactive multispectral optoacoustic tomographic vs lymphoscintigraphic imaging for biopsy of sentinel lymph nodes in patients with melanoma.

## Introduction

Knowledge of regional lymph node status is important in cancer staging because it determines the prognosis of patients and informs further treatment. Therefore, sentinel lymph node biopsy (SLNB) is routinely performed in many malignant tumors, including melanoma,^1^ as well as breast,^2^ vulvar,^3^ penile,^4^ Merkel cell,^5^ squamous cell,^6^ and sweat gland^7^ carcinomas. In addition, SLNB is partially used in esophageal,^8^ gastric,^9^ and colorectal^10^ carcinomas. The conventional standard for SLN detection is lymphoscintigraphic imaging with technetium Tc 99m.^11,12,13^

There are known disadvantages of Tc 99m use. Lymphoscintigraphic imaging has poor spatial resolution (approximately 20 mm) and insufficient precision for SLN localization with a single projected image.^14^ The involvement of radioisotopes is expensive and creates logistical challenges, including isotope handling, staff training, and legislative requirements.^15^ Furthermore, Tc 99m represents a radioactive burden for patients and surgical personnel. In addition, there is a worldwide shortage of Tc 99m.^16,17^ These shortcomings could be avoided by implementing alternative, preferably nonradioactive, imaging techniques.

Indocyanine green (ICG) is a nonradioactive dye that can be applied for SLN labeling and detection. Indocyanine green has been widely used for staging breast and gastrointestinal cancers when lymphatic drainage patterns are predictable.^18,19^ However, in patients with melanoma and unpredictable lymphatic drainage, ICG detected with near-infrared (NIR) cameras proved to be inferior to Tc 99m in defining lymphatic basins containing metastases owing to the limited tissue penetration of NIR cameras (1.0-1.5 cm).^7,20,21^

**Figure 1.**
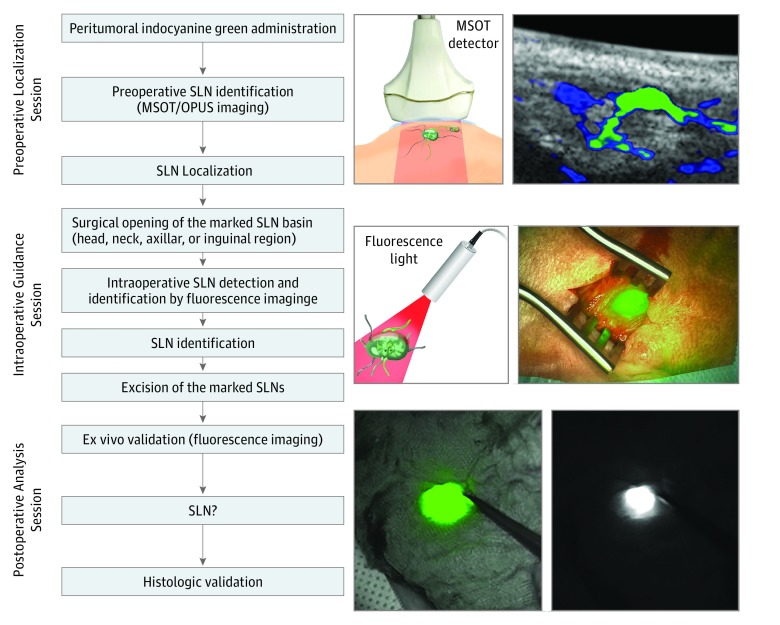
Flowchart of Proposed Sentinel Lymph Node (SLN) Biopsy Guidance Using Trimodal Imaging Preoperative imaging with multispectral optoacoustic tomography (MSOT) and optoacoustic ultrasonography (OPUS), followed by intraoperative imaging with a near-infrared camera system, followed by postoperative validation of in vivo signals.

Multispectral optoacoustic tomographic (MSOT) imaging could help to overcome the above-mentioned difficulties by simultaneously providing optical molecular contrast with deep tissue imaging (up to 5 cm) of ICG-labeled SLNs.^22,23,24,25,26^ This advanced technique has led to preoperative SLN mapping by means of ICG and MSOT imaging gaining in popularity.^26^ The goal of our study was to assess the concordance between ICG-labeled SLNs detected via MSOT imaging and conventional Tc 99m–labeled SLNs to determine whether nonradioactive MSOT imaging–based SLN detection can be used alone in tumors with unpredictable lymphatic drainage, such as melanoma (Figure 1).

## Methods

### Study Design

The study objective was to investigate the extent to which optoacoustic technique, in combination with ICG injection, provides a reliable, nonradioactive method for accurate SLN detection in patients with melanoma. The primary end point was the concordance of SLNs identified by lymphoscintigraphic imaging with those identified by MSOT imaging and ICG. This study was approved by the institutional review board at the University Hospital-Essen and registered at the German Clinical Trials Register. All patients provided written informed consent according to the Declaration of Helsinki^27^; there was no financial compensation. This study followed the Strengthening the Reporting of Observational Studies in Epidemiology (STROBE) reporting guideline for cross-sectional studies.

To estimate the number of SLNs that needed to be examined, we assumed a detection rate of 100% for the conventional standard Tc 99m method, a discordance rate between the conventional standard Tc 99m method and MSOT imaging plus ICG of 6%,^28^ and set δ level at 5%. We found that 168 SLNs (approximately 70 patients: 2.4 SLNs per patient) needed to be examined to demonstrate equivalence between the 2 methods with 90% power and type I error (α) of 5% (eAppendix 1 in the Supplement).^29,30^ Based on this calculation, we aimed to include 83 patients in this cross-sectional study.

### Participant Population

Participants were recruited from the patient population attending the Department of Dermatology at the University Hospital-Essen. Eligibility criteria included age 18 years or older, capability of providing written informed consent, and newly diagnosed melanoma in *American Joint Committee on Cancer, 7th edition*, stages I and II (>pT1b).^31^ Participants must have had clinically negative lymph nodes, confirmed by clinical examination and ultrasonographic evaluation.^32^ Exclusions included contraindications for surgery, history of allergic reactions attributed to ICG, and uncontrolled intercurrent illness including, but not limited to, ongoing or active infection of the surgical field, symptomatic congestive heart failure, unstable angina pectoris, cardiac arrhythmia, or psychiatric illness or social situations that would limit adherence to study requirements.

### Imaging

A total of 80 MBq of Tc 99m nanocolloid was injected around the tumor in 4 intradermal deposits of 0.1 mL. Dynamic images of the corresponding anatomic region were acquired at 30 seconds per frame for 5 minutes with a total of 10 frames. Subsequently, anterior, lateral, and oblique projections were acquired for 5 minutes each, using a dual-detector γ camera with a mounted, 2-row multidetector computed tomographic (CT) scanner (Symbia T; Siemens Healthcare). Single-photon emission CT/CT (SPECT/CT) images of the region in which an SLN was visualized were obtained immediately after the planar images showing an SLN. The reconstructed data were displayed as sagittal, coronal, and axial slices. Inherent image fusions were generated from the coregistered SPECT and low-dose CT images using the E.soft 2007 application package (Siemens Healthcare). The surgeons were blinded to the findings of the preoperative lymphoscintigraphic and SPECT/CT images.

### SLN Biopsy

Sentinel lymph node biopsy was performed as a standard procedure according to the guidelines of the Deutsche Dermatologische Gesellschaft (German Association of Dermatology),^33^ as previously described.^13^

In many publications, the concordance rate of ICG and Tc 99m is artificially inflated because the Tc 99m label enhances the ability to visualize ICG.^18,20,28,34^ To circumvent this bias, our surgeons were blinded to the lymphoscintigraphic imaging results in the beginning of the SLNB procedure. In addition, the use of a γ probe was restricted until the SLNB procedure was performed using the nonradioactive MSOT-imaging techniques and ICG dye alone.

All excised ICG-labeled SLNs were tested for radioactivity using a γ probe and classified as hot (Tc 99m positive) or cold (Tc 99m negative). After finishing the nonradioactive SLNB, SPECT/CT imaging and lymphoscintigraphic imaging results were used to identify potential additive SLN basins. All SLN basins were then checked with a γ probe to determine significant residual radioactivity. Any Tc 99m–positive SLNs that had not been identified were excised using a γ probe.

### Indocyanine Green Dye

Indocyanine green is a member of the cyanine dyes, has a green color, and is a powderlike solid substance at 25 °C. The absorption maximum is 697 nm in water at a concentration of 0.5 g/L and 779 nm at 0.0005 g/L. After injection, ICG binds almost completely to globulins, preferentially to α-lipoproteins, within 1 to 2 seconds. The use of ICG in SLN detection in stomach, breast,^35,36^ ovarian,^37^ and cutaneous malignant tumors^21,38^ has been reported.^7,20^

### MSOT Imaging

Preoperatively, 1 mL (0.5 mg) of ICG solution was injected peritumorally. Lymphatic drainage was observed in real time by MSOT imaging. A commercially available handheld MSOT imaging system was used (Acuity Echo; iThera Medical GmbH). Nanosecond excitation laser pulses were generated at a repetition rate of 25 Hz in a wavelength range of 680 to 900 nm. For multispectral imaging, 6 wavelengths (700, 730, 760, 800, 850, and 900 nm) were selected to allow separation of chromophores by spectral unmixing. A cylindrically focused, 256-element detector array (center frequency, 4 MHz; send/receive bandwidth, 52%; resolution, approximately 190 μm) and 135° coverage provided 2-dimensional cross-sectional images with a field of view of 40 × 40 mm^2^ and a pixel size of 62.5 μm. Laser light was delivered via a fiber bundle (CeramOptec GmbH). MSOT imaging was used without the MSOT investigators’ prior knowledge of the SLN-containing regions (Figure 1). The results of lymphoscintigraphic imaging were not applied to detect SLN by MSOT imaging.

### Image Creation

Optoacoustic signals acquired by the aforementioned system were tomographically reconstructed using standard backprojection.^39^ Multispectral illumination was used at the described wavelengths to separate individual contributions of absorbers. With use of linear regression spectral unmixing, known absorption spectra of the expected absorber (ICG) were inverted and multiplied with the acquired images to reveal the spatial distribution of the absorber in separate component images.^40^

### Hybrid Optoacoustic-Ultrasonographic Imaging

The described 2-dimensional detector generated a pulse-echo ultrasonographic image using an integrated ultrasonographic imaging platform described elsewhere.^41^ Interleaved acquisition of optoacoustic and ultrasonographic images was facilitated through a custom-made, laser-triggered multiplexer unit connected to the transducer array that ensures switching between 2 imaging modes. For generation of the pulse-echo ultrasonographic images, the synthetic transmit aperture technique^42^ was used with a single element sequentially transmitting a pulse (40 V peak to peak, 6 MHz) and a subaperture of 128 elements (one-half of the full aperture) receiving the resulting echoes. The data collected by 2 subapertures, covering the full view angle of the handheld array, were then combined to form a final image using a spatial compounding technique at a field of view of 40 × 40 mm^2^ with a pixel size of 180 μm.

### Intraoperative NIR Fluorescence Imaging

Intraoperative ICG visualization was performed applying the Quest Spectrum Platform (Quest Medical Imaging). This custom camera allows simultaneous capture of color and NIR images. For open procedures, the ring light was attached to the camera, which contains a wide field lens and an 8-piece ring that is connected by optical fibers to the light engine. Further details are presented in the eAppendix 1 in the Supplement. 

### False-negative Sentinel Node

The SLN excision was considered false-negative if a recurrence developed within 12 months after SLN excision in the basin from which a tumor-free SLN had been removed.^43^ The false-negative rate is the ratio of false-negative results to the total number of positive nodes (false-negatives plus true-positives).^44^

### Statistical Analysis

Differences in patient characteristics were evaluated with the Wilcoxon-Mann-Whitney test for continuous variables and the χ^2^ test or Fisher exact test, the latter when expected cell frequencies were low, for categorical variables.

The primary end point, the SLN basins and SLN concordance of ICG with Tc 99m, was defined as the number of preoperative Tc 99m–marked SLN basins and SLNs that were detected by ICG, divided by the number of Tc 99m–marked SLN basins and SLNs. A secondary end point was the per-patient concordance rate, defined as the percentage of patients for whom all nodes detected by Tc 99m were detected by ICG. Another secondary end point, the reverse concordance, evaluated the proportion of all nodes detected by ICG that were also Tc 99m positive. Differences were regarded as statistically significant at *P* < .05 with 2-tailed, unpaired testing. The statistical analysis was performed with SPSS, version 20 (SPSS Inc).

## Results

### Patient Characteristics

Sentinel lymph node biopsies were analyzed in 83 patients between June 2, 2014, and February 22, 2019. Characteristics are depicted in Table 1. The mean (SD) age of the sample was 54.61 (17.53) years; 47 were men (56.6%) and 36 were women (43.4%). The mean (SD) melanoma tumor depth was 2.93 (3.3) mm.

**Table 1.** Patient Characteristics

Characteristic	No. (%)
Patients	83
Age, y	
Mean (SD)	54.61 (17.53)
Median (range)	57.50 (21-85)
Sex, No. (%)	
Male	47 (56.6)
Female	36 (43.4)
Tumor depth, mm	
Mean (SD)	2.93 (3.37)
Median (range)	1.70 (0.8-21.0)
Localization of primary tumor	
Head/neck	4 (4.82)
Trunk	35 (42.17)
Upper extremity	18 (21.69)
Lower extremity	26 (31.32)
BMI	
Mean (SD)	26.8 (5.56)
Normal, healthy weight	38 (45.8)
Overweight	29 (34.9)
Obese class I, moderate	11 (13.3)
Obese class II, severe	3 (3.6)
Obese class III, very severe	2 (2.4)
Ulceration	30 (36.14)
Localization of SLN	169 (100)
Head/neck	5 (2.96)
Axilla	101 (59.75)
Groin	59 (34.92)
Popliteal region	4 (2.37)
SLN basins per patient	83 (100)
1 Localization	58 (69.88)
2 Localizations	19 (22.89)
3 Localizations	4 (4.82)
4 Localizations	2 (2.41)
Excised lymph nodes	
SLNs per patient, mean (SD) [No. of SLNs/No. of patients]	2.04 (1.20) [169/83]
SLNs per patient, median (range)	2.00 (1-5)^a^
SLN positive, No./total No. excised (%)	30/169 (17.75)
Patients with positive SLNs, No./total No. (%)	23/88 (27.71)

Abbreviations: BMI, body mass index (calculated as weight in kilograms divided by height in meters squared); SLN, sentinel lymph node; SLNE, SLN excision.

^a^ The SLNE was performed in up to 3 anatomic localizations (head and neck, axilla, and groin).

### Preoperative SLN Identification

**Figure 2.**
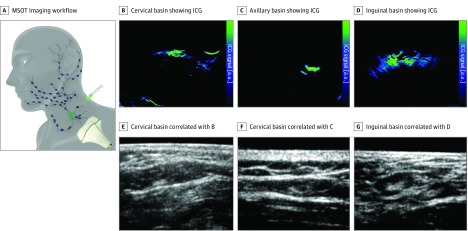
Sentinel Lymph Node Image by Basin A, Schematic showing handheld multispectral optoacoustic tomographic (MSOT) imaging workflow. B, Cervical basin of MSOT image showing indocyanine green (ICG). C, Axillary basin of MSOT image showing ICG. D, Inguinal basin showing ICG. E, Cervical basin showing ultrasonographic image correlating with panel B. F, Axillary basin showing ultrasonographic image correlating with panel C. G, Inguinal basin showing ultrasonographic image correlating with panel D. a.u. indicates auxiliary unit.

We administered ICG peritumorally and used a 2-dimensional MSOT detector to image SLNs (Figure 2). The primary study end point was defined as the number of preoperative Tc 99m–marked SLN basins and SLNs that were detected with ICG by MSOT with optoacoustic-ultrasonographic (OPUS) imaging. Preoperatively, 116 SLN basins were detected by lymphoscintigraphic plus SPECT/CT imaging, 106 by MSOT/OPUS imaging, and ICG. In 4 of the 10 missing cases, an additional single SLN was found in the popliteal region. This region was not investigated with MSOT imaging but was visible in the SPECT/CT image. Since the region was not scanned by MSOT, these 3 SLNs and SLN basins were excluded from the comparative analysis. In these cases, an inguinal SLN was also marked. In the missing 6 cases (all with a melanoma on the patient’s trunk), an additional axillary SLN basin was not detected by MSOT imaging. Therefore, the 2 methods were concordant for 106 of 112 SLN basins (concordance rate, 94.6%).

Intraoperatively, 165 Tc 99m–labeled SLNs were detected using a γ probe (popliteal region excluded) and excised; preoperative MSOT/OPUS imaging plus intraoperative NIR camera detected 159 of all found SLNs identified (Table 2). The 2 methods were concordant for 159 of 165 SLNs (96.4%). Six of 165 SLNs (3.6%) were preoperatively detected by Tc 99m and not by ICG with MSOT/OPUS imaging (95% CI, 2.6%-8.8%, with application of the Lu and Bean algorithm^45^). Since this 95% CI does not include −5% (ie, in relation to equivalence margins, −5% and +5%), we were able to reject a 1-sided null hypothesis A and conclude that ICG with MSOT imaging was not inferior to Tc 99m.

**Table 2.** Performance of In Vivo MSOT Imaging in Comparison With Lymphoscintigraphy Plus SPECT/CT in SLN Basin Identification

Variable	No. (%)
Preoperative SLN basin identifications	
Lymphoscintigraphy plus SPECT/CT, No.	116
Lymphoscintigraphy plus SPECT/CT without popliteal region, No. (%)^a^	112 (100)
MSOT plus ICG, No. (%)	84 (75.0)
MSOT/OPUS plus ICG, No. (%)	106 (94.6)
Intraoperative SLN identifications	
Use of γ probe after lymphoscintigraphy plus SPECT/CT, No./total No. (%)	169/169 (100)
Use of γ probe after lymphoscintigraphy plus SPECT/CT without popliteal region, No./total No. (%)^a^	165/165 (100)
MSOT/OPUS plus fluorescence camera plus ICG, No./total No. (%)	159/165 (96.4)

Abbrevations: ICG, indocyanine green; MSOT, multispectral optoacoustic tomography; OPUS, optoacoustic ultrasound; SLN, sentinel lymph node; SPECT/CT, single-photon emission computed tomographic or computed tomographic imaging.

^a^ In 4 missing cases there was an additional single SLN in the popliteal region. This region was not investigated with MSOT but was visible on lymphoscintigraphy plus SPECT/CT. Since the region was not scanned by MSOT, these 4 SLNs were excluded from the comparative analysis.

All 83 patients with at least 1 Tc 99m–marked SLN also had at least 1 of their radioactive marked nodes fluorescently labeled, resulting in a per-patient concordance rate of 100%. Reverse concordance was defined as the percentage of fluorescent SLNs that were also Tc 99m positive. There were 163 fluorescent SLNs identified, and all were Tc 99m marked. Similarly, in 81 of 81 patients with at least 1 ICG-positive node, all of those nodes were also Tc 99m positive.

In the inguinal SLN example, there was a strong ICG signal in the SLNs at 10 mm below the skin surface (Figure 2). For the cervical SLNs, the signal was seen at a depth of approximately 7 mm (Figure 2). The localized signal of the axillary SLN example was at a depth of approximately 20 mm (Figure 2).

Pulse-echo ultrasonographic images of the SLNs were taken using the 2-dimensional MSOT image detector in combination with an integrated ultrasonographic imaging device that was evaluated to further improve SLN detection. The specific ICG signal simultaneously acquired by MSOT imaging could then be overlaid onto the ultrasonographic image. With MSOT imaging alone, we identified 84 of 110 ICG-labeled SLN basins (76.4%). In 22 more basins, we used an integrated OPUS, which simplified and improved SLN detection (106 of 112 [94.6%]) (Figure 2).

### Follow-up

Patient follow-up was between 1 and 42 months (mean [SD], 26.7 [10.47] months; median [range], 29.0 [2-42] months). The false-negative SLN rate was 0%. Eight of 83 patients (9.6%) developed distant metastasis (liver metastasis, 3; brain metastasis, 2; lung metastasis, 2; and bone metastasis, 1) and 4 patients (4.8%) showed subcutaneous in-transit metastasis during follow-up.

### Adverse Events

There were no study-related serious adverse events. Intradermal ICG injection caused no local skin changes, allergic reactions, or permanent skin tattoos.

## Discussion

To date, the sole application of fluorescent dyes for SLN labeling has not gained international acceptance because nonradioactive-marked SLNs were almost exclusively detected by NIR cameras having an insufficient limited penetration depth of 1 to 1.5 cm.^20,46,47^ To further increase depth sensitivity, we used MSOT imaging preoperatively to detect SLN basins and SLNs. To our knowledge, this is the first adequately powered (90% power) prospective study performed to analyze the benefit of nonradioactive SLN detection in comparison with the conventional standard radiotracer lymphoscintigraphic plus SPECT/CT imaging. The comparison was designed to analyze whether the MSOT imaging-based ICG method is a reliable alternative to the radiotracer method. Our study suggests for the first time that nonradioactive MSOT imaging-based SLN detection is comparable to Tc 99m nanocolloid–guided SLN detection in all anatomic regions (Table 1).^26^

Compared with the depth limitations of ICG detection by NIR cameras^20,46,47^ (eFigure in the Supplement), MSOT imaging can pick up ICG-marked SLNs at a sufficient depth of up to 5 cm (determined by SPECT/CT imaging).^26^ Preoperatively, with MSOT imaging alone, we identified 84 ICG-labeled SLN basins. In 22 more basins, we had to use an integrated OPUS, which simplified and improved SLN basins and SLN detection (Figure 2).^26,48^ By combining optoacoustic sensitivity with ultrasonographic imaging, we obtained lymph node characterization and morphologic findings simultaneously. In the clinic, this process currently consists of 2 steps: initial preoperative ultrasonographic imaging followed by radioactive labeling of the SLN. Therefore, in comparison with the conventional standard (radioactive lymphoscintigraphic and SPECT/CT imaging), we identified preoperatively 106 of 112 SLN basins and achieved a concordance rate of 94.6%. Intraoperatively, 159 of 165 SLNs (96.4%) were identified by nonradioactive imaging. All 6 missed SLNs were only labeled radioactively. Most likely, the distance of the Tc 99m injections by the nuclear medicine physicians to the primary tumor was greater than the distance of the ICG injections by the surgeons to the primary tumor, resulting in additive afferent lymphatics and lymph nodes being labeled radioactively that were not true SLNs. This is supported by the fact that no positive SLN was missed using the nonradioactive technique alone.

The results from this study are a step toward clinical implementation of optoacoustic imaging for nonradioactive SLN detection in malignant lesions with lymphatic spread. Sentinel lymph node detection by lymphoscintigraphic imaging is difficult in complex regions, such as the head and neck, owing to the high density of closely grouped lymphatic structures. The close proximity of the primary tumor to the SLN can result in lymph nodes being masked by the injection site of the radionuclide, resulting in higher false-negative rates in SLNBs. In some cases, this problem can be circumvented by repositioning the patient and obtaining a different view of the region of interest. With MSOT imaging, however, localization of ICG signals is simplified with use of a handheld detector placed in a location of interest (eg, avoiding the injection site). Tomographic detection can localize signals without the need for repositioning. Furthermore, the feedback from MSOT imaging is real time and performed by the clinician; thus, the patient can be assessed immediately before surgery in the position in which surgery will occur.

**Figure 3.**
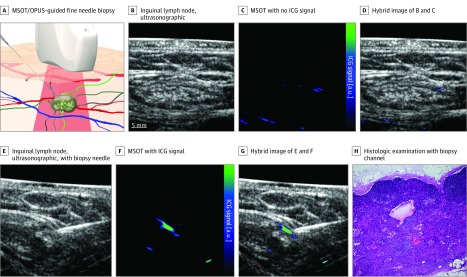
Optoacoustic-Guided Fine-Needle Biopsy of Sentinel Lymph Node A, Schematic illustration of a multispectral optoacoustic tomography (MSOT)/optoacoustic ultrasonographic (OPUS)–guided fine-needle biopsy. B, Inguinal lymph node, ultrasonographic image, C, MSOT image correlating with panel A showing no indocyanine green (ICG) signal. D, Hybrid image of panels B and C. E, Inguinal lymph node, ultrasonographic image, with biopsy needle. F, MSOT image showing ICG, correlating with panel E. G, Hybrid image of panels E and F. H, Lymph node histologic examination (hematoxylin-eosin staining; original magnification x40) with biopsy channel. a.u. indicates auxiliary unit.

This novel strategy may also be used to perform fine-needle biopsies (FNBs) of the SLNs in various malignant tumors.^49^ For FNBs of the SLNs, MSOT/OPUS imaging might serve as a real-time modality to first locate the SLN and then guide an FNB. Figure 3 shows an example of the corresponding MSOT and OPUS images revealing the SLN and the needle, respectively. The contrast from the needle in the MSOT image is superior compared with the corresponding OPUS image owing to the speckle-free nature of the OPUS image^50^ and its improved angular sensitivity compared with conventional ultrasonographic imaging.^51^ The coregistered MSOT and OPUS image shows the capability of the technology to locate lymph nodes via anatomic features in OPUS imaging and verify the lymph node as an SLN using MSOT imaging. In addition, these results suggest feasibility of optoacoustic-guided, minimally invasive FNBs of SLNs, thereby giving the possibility to combine FNBs of SLNs with ultrasonographic image morphologic criteria as well as with molecular staging methods as previously described for melanoma^49,52,53^ and breast,^54^ thyroid,^55^ lung,^56^ and pancreatic^57^ cancer.

Regarding costs, eliminating preoperative lymphoscintigraphic imaging can reduce inpatient hospital time and material costs. Indocyanine green is readily available, easy to store, and less costly than radioactive tracers (eAppendix 2 and eTable in the Supplement).

Blue dyes, such as methylene blue, were not used because of potential allergic reactions and the risk of permanent tattoos.^58^ Nevertheless, MSOT imaging technology and the applied intraoperative NIR camera system can also visualize methylene blue.

### Limitations

This study has limitations. First, 4 SLN basins of patients with missing data (popliteal region was not scanned) had to be excluded from our analysis. Second, the monocentric and cross-sectional nature of this study resulted in limited descriptive statistical analyses. Therefore, a multicenter, randomized prospective trial is envisioned. Third, cost variations between different countries and health systems limit the generalizability of the findings. Regarding financial aspects, the main factors associated with reductions in cost in nonradioactive MSOT imaging apply independent of national circumstances. Shortening inpatient hospital time could be beneficial irrespective of the health system. Our results thus suggest medical and economic advantages for incorporating a MSOT imaging strategy into the management of patients with cancer.

## Conclusions

Our findings suggest that MSOT-based ICG imaging can be performed independent of conventional SLN labeling. Use of MSOT may possibly eliminate the need for radioactive tracers.

## Notes

Supplement. **eAppendix 1.** Supplementary Methods **eAppendix 2.** Costs **eTable.** Costs of SLNB **eFigure.** Axillary LN Detection: ICG Injection and Limitations of ICG
