# Supplementary material for: Assessment of Nonradioactive Multispectral Optoacoustic Tomographic Imaging With Conventional Lymphoscintigraphic Imaging for Sentinel Lymph Node Biopsy in Melanoma
Source: JAMA Netw Open. 2019 Aug 14;2(8):e199020. doi: 10.1001/jamanetworkopen.2019.9020 (PMC6694392; doi:10.1001/jamanetworkopen.2019.9020)
Supplement: Supplement. — eAppendix 1. Supplementary Methods eAppendix 2. Costs eTable. Costs of SLNB eFigure. Axillary LN Detection: ICG Injection and Limitations of ICG [file jamanetwopen-2-e199020-s001.pdf]

Supplementary Online Content

Stoffels I, Jansen P, Petri M, et al. Assessment of nonradioactive multispectral optoacoustic tomographic imaging with conventional lymphoscintigraphic imaging for sentinel lymph node biopsy in melanoma. *JAMA Netw Open*. 2019;2(8):e199020. doi:10.1001/jamanetworkopen.2019.9020

eAppendix 1. Supplementary Methods

eAppendix 2. Costs

eTable. Costs of SLNB

eFigure. Axillary LN Detection: ICG Injection and Limitations of ICG

This supplementary material has been provided by the authors to give readers additional information about their work.

© 2019 Stoffels I et al. *JAMA Network Open.*

eAppendix 1. Supplementary Methods

Indocyanine green

Shortly before MSOT imaging, a vial of 25-mg ICG (Pulsion Medical Systems AG) was dissolved in 50 ml of water for injection (B. Braun Medical), yielding a concentration of 0.5 mg/ml. One milliliter of dissolved ICG was injected peritumorally 30 min before MSOT imaging.

Intraoperative NIR Fluorescence Imaging

Intraoperative ICG visualization was performed by using the Quest Spectrum™ Platform (part number: ART-SYS-0020 R0 and software: Capture Suite v1.4.7; Quest Medical imaging, Netherlands) which has a custom camera that allows simultaneous capturing of color images and up to two NIR images. For open procedures, ring light was attached to the camera, which contains a wide field lens and an eight-piece ring that is connected by optical fibers to the light engine.

The NIR-System was used to generate white light by using LEDs and NIR light, for fluorescent imaging, by using lasers. The two lasers can generate light with peak intensities of approximately 700 and 800 nm. Reflected excitation light is blocked by filters. Subsequently, the light passes through a lens that could be used for focusing. The light then enters a prism containing a dichroic coating in order to separate the visible and the two NIR lights. The light beams are captured by a CCD sensor. For visualization of ICG, the 800 nm laser was selected. The output of the light engine can be controlled with the software to assure optimal visualization or used in auto settings. Exposure times and sensor gains were separately adjusted for both imaging channels, and acquisition was

© 2019 Stoffels I et al. *JAMA Network Open.*

synchronized to the longest exposure time. The raw data of both sensors could be saved as individual snapshots or as a real-time movie. During procedures, the visible light channel, the NIR fluorescence channel, and an adjustable overlay are presented.

© 2019 Stoffels I et al. *JAMA Network Open.*

eAppendix 2. Costs

At the University Hospital of Essen-Duisburg the variable costs for a SLNB in an operating room were \$ 9.44/min. The fixed costs for infrastructure and material per SLNE in an operating room were \$ 98.22. A SLNB procedure costs on average (mean) \$ 614.79. The additional cost for lymphoscintigraphy of \$ 151.52 and SPECT/CT of \$ 135.87 were based on the “Deutsche Krankenhausgesellschaft Normaltarif”. The costs for technetium and ICG are different (\$ 79.25/patient vs. \$ 6.73/patient, respectively) (Tab. 3). The acquisition costs for a SPECT/CT are approximately \$ 350.000 compared to the expected price of approximately \$ 500.000 for an MSOT Acuity system once it is certified as a medical device.

Aside from the use of lymphosctintigraphy with SPECT/CT or MSOT/US, the cost difference is mostly explained by differences in hospital stay. The hospital stay includes the admission to the dermatology ward and pre- and post-op nursing care (\$ 304.63/day, Tab. 3). However, the use of technetium requires a specialist in nuclear medicine with appropriate premises, personnel equipment and an additional in-patient day. Irrespective of this, a radioactive-free marking would be preferable to a radioactive technique.

© 2019 Stoffels I et al. *JAMA Network Open.*

eTable. Costs of SLNB

Listing of costs for inpatient SLNB with nuclear medicine label. Costs per patient in € (Germany)

	**Unit costs (€)**	
**Lymphoscintigraphy**	130.00	
**SPECT/CT (€)**	116.57	
**Hospital stay (€/day)**	261.36	
**Costs operation (€)**		
**- mean**		522.83
**-standard deviation**		282.22
**Costs hospital stay (€)**		
**- mean**		1810.85
**- standard deviation**		1132.65
**Total (€)**		
**- mean**		2450.25
**- standard deviation**		1415.79
**Total (€)**		
**- mean**		2121.18
**- standard deviation**		1397.68

© 2019 Stoffels I et al. *JAMA Network Open.*

eFigure. Axillary LN detection: ICG injection and limitations of ICG

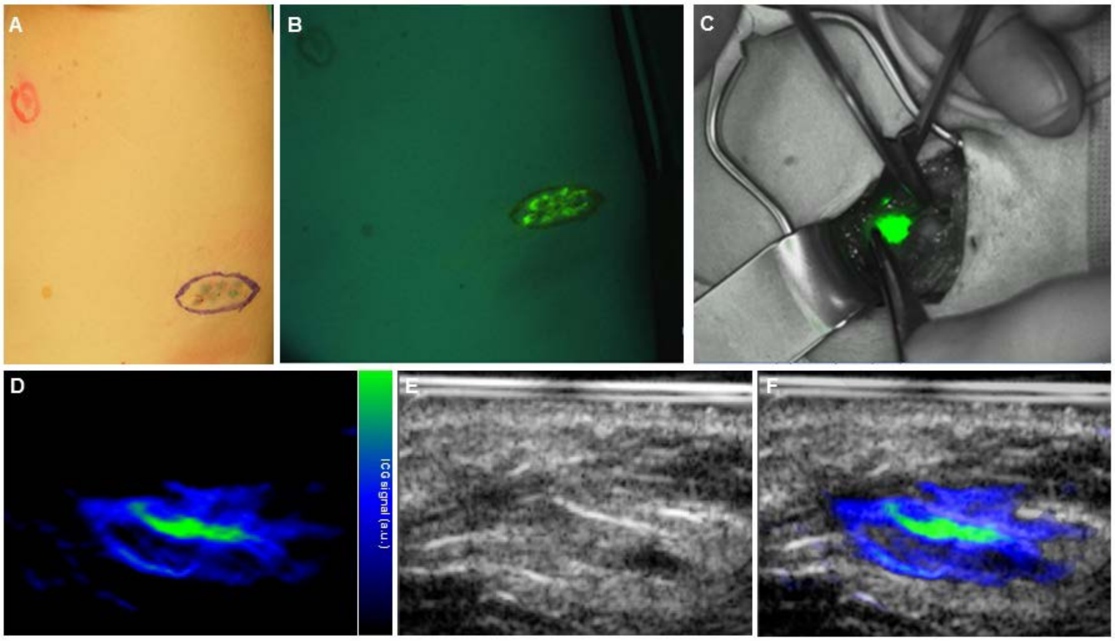

A: Patient with a malignancy on the left trunk. ICG was injected intradermally in the region of the primary tumor. B: Preoperative mapping of lymphatic drainage with ICG, showing no lymphatic vessels detectable by NIR-camera. C: Intraoperative planar fluorescence image showing the fluorescent SLN in the left axilla. D: Preoperative MSOT image showing SLN in the left axilla. E: OPUS image corresponding to the MSOT image in panel D. E: Hybrid image of D+E.

Abbrevations: LN, lymph node; ICG, indocyaningreen; NIR-camera, near-infrared camera; MSOT, multispectral optoacoustic tomography; SLN, sentinel lymph node; OPUS, optoacoustic ultrasound

© 2019 Stoffels I et al. *JAMA Network Open.*
